# How Do You Solve a Problem like *Letharia*? A New Look at Cryptic Species in Lichen-Forming Fungi Using Bayesian Clustering and SNPs from Multilocus Sequence Data

**DOI:** 10.1371/journal.pone.0097556

**Published:** 2014-05-15

**Authors:** Susanne Altermann, Steven D. Leavitt, Trevor Goward, Matthew P. Nelsen, H. Thorsten Lumbsch

**Affiliations:** 1 Biology Department, Whitman College, Walla Walla, Washington, United States of America; 2 Committee on Evolutionary Biology, University of Chicago, Chicago, Illinois, United States of America; 3 Science and Education, The Field Museum, Chicago, Illinois, United States of America; 4 Beaty Biodiversity Museum, University of British Columbia, Vancouver, British Columbia, Canada; University of Guelph, Canada

## Abstract

The inclusion of molecular data is increasingly an integral part of studies assessing species boundaries. Analyses based on predefined groups may obscure patterns of differentiation, and population assignment tests provide an alternative for identifying population structure and barriers to gene flow. In this study, we apply population assignment tests implemented in the programs STRUCTURE and BAPS to single nucleotide polymorphisms from DNA sequence data generated for three previous studies of the lichenized fungal genus *Letharia*. Previous molecular work employing a gene genealogical approach circumscribed six species-level lineages within the genus, four putative lineages within the nominal taxon *L. columbiana* (Nutt.) J.W. Thomson and two sorediate lineages. We show that Bayesian clustering implemented in the program STRUCTURE was generally able to recover the same six putative *Letharia* lineages. Population assignments were largely consistent across a range of scenarios, including: extensive amounts of missing data, the exclusion of SNPs from variable markers, and inferences based on SNPs from as few as three gene regions. While our study provided additional evidence corroborating the six candidate *Letharia* species, the equivalence of these genetic clusters with species-level lineages is uncertain due, in part, to limited phylogenetic signal. Furthermore, both the BAPS analysis and the ad hoc Δ*K* statistic from results of the STRUCTURE analysis suggest that population structure can possibly be captured with fewer genetic groups. Our findings also suggest that uneven sampling across taxa may be responsible for the contrasting inferences of population substructure. Our results consistently supported two distinct sorediate groups, ‘*L. lupina*’ and *L. vulpina*, and subtle morphological differences support this distinction. Similarly, the putative apotheciate species ‘*L. lucida*’ was also consistently supported as a distinct genetic cluster. However, additional studies will be required to elucidate the relationships of other *L. columbiana* s.l. populations with the two sorediate genetic clusters.

## Introduction

Our ability to accurately recognize species-level biological diversity is key to generating and testing many ecological and evolutionary hypotheses [1]. However, recognizing species boundaries is notoriously challenging and controversial, especially in groups with recent and rapid diversification histories. The greatest confidence in species delimitations is attained using independent sources of data (i.e. ecology, behavior, morphology, genetic, etc.) and multiple analytical approaches [2]–[5]. The general lineage concept, equating species to segments of separately evolving metapopulation lineages, provides a framework for species delimitation using a variety of operational criteria, data sets, and analytical methods to detect evidence for lineage independence (reproductive isolation, morphological, ecological, and/or genetic differences, etc.) [6]–[8]. Multilocus sequence data can provide unprecedented and accurate insights into species delimitation and the processes of speciation [9]–[13]. The inclusion of molecular data is now considered an integral part of many studies assessing species boundaries, and novel analytic approaches continue to be developed to assess the increasing availability of genetic data [14].

In many cases, the focus of species delimitation problems exists at the interface of traditional population genetic and phylogenetic analyses [2], [10], [11]. Incomplete lineage sorting, recent or ongoing gene flow, and ambiguity in specimen identification or population assignment may confound accurate recognition of species boundaries and evolutionary histories in groups with recent diversification [10], [15]. In spite of the inherent challenges to the recognition of species boundaries in recently diverged complexes, these groups can provide important insights into the factors that drive diversification (e.g. [11], [16], [17]).

**Figure 1:**
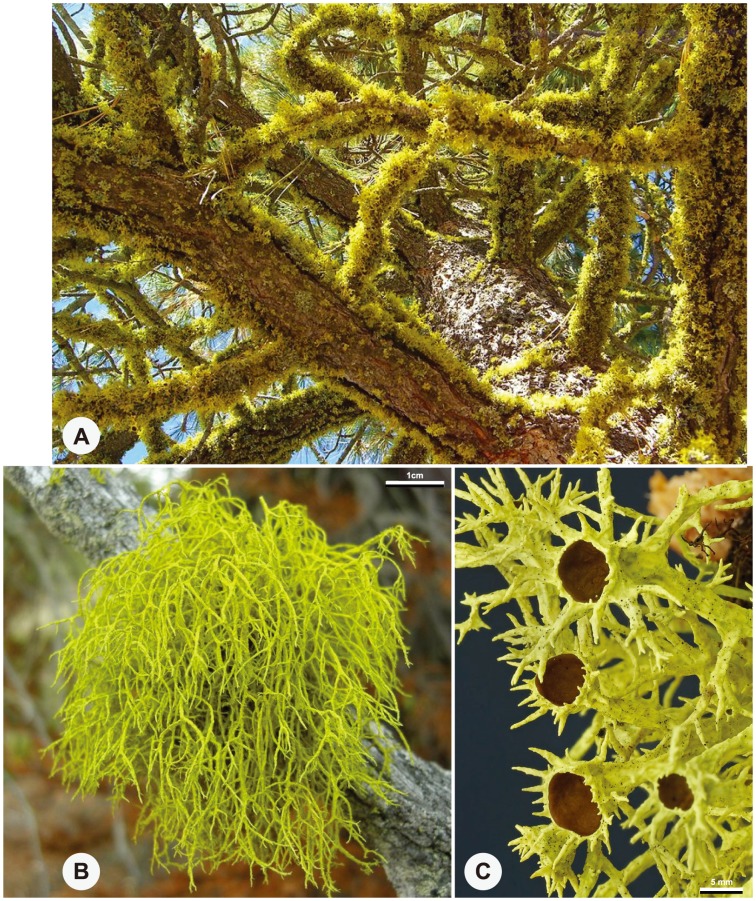
Morphological variation in *Letharia*. A. *L. vulpina* population on *Pinus jeffreyi* in the Pine Mountains in southern California, USA (photo credit: Jason Hollinger). B. Habit shot of *L*. ‘*lupina*’ on dead *Pinus* branch, Jackman Flats, British Columbia, Canada (photo credit: Jason Hollinger). C. Habit shot of *L. columbiana* s.l. on *Pinus ponderosa* in the eastern Cascades, Washington, USA (photo credit: Richard Droker).

In this study we investigated species boundaries in the lichen-forming fungal genus *Letharia* (Parmeliaceae) (Th. Fr.) Zahlbr. Previous research suggests a recent diversification history, characterized by limited genetic variation across the genus and patterns of incomplete lineage sorting in multiple loci [18]. *Letharia* populations comprise a conspicuous component of some epiphytic communities in the montane forests of western North America (Fig. 1). The genus also occurs in continental Europe, northern Africa, Cyprus and the Caucasus, although it less common in the Old World and appears to be declining in Northern Europe [19]–[21]. Traditionally, *Letharia* was thought to include two distinct species, *L. columbiana* (Nutt.) J. W. Thomson and *L. vulpina* (L.) Hue, representing a classic example of a lichen species pair [22], [23]. In this scheme, *L. vulpina* is recognized by the production of asexual reproductive structures – soredia and/or isidioid soredia – which contain both fungal and algal cells (Fig. 1B), while *L. columbiana* has abundant ascomata (sexual reproductive structures) instead (Fig. 1C). However, some *L. columbiana* individuals also produce vegetative diaspores in the form of isidia, whereas *L. vulpina* can on occasion bear apothecia [18], [19]; and evidence of recombination through sexual reproduction has been documented for typically sorediate forms [24]. Reproductive and dispersal barriers among closely related *Letharia* species remain uncertain. While *L. vulpina* is geographically widespread and occurs in both the New and Old Worlds, *L. columbiana* is restricted to western North America [19], [20].

Using a phylogenetic species recognition (PSR) approach, Kroken and Taylor [18] proposed that *Letharia* was comprised of six species-level lineages, rather than the two traditionally accepted species. This iconic study played a major role in initiating molecular approaches for recognizing species boundaries in non-model organisms, and particularly lichen-forming fungi (reviewed in [25]–[27]). Although *Letharia* represents one of the best-studied lichenized fungal genera [18]–[20], [24], [28], only one of the previously unrecognized lineages circumscribed using molecular data has been formally described, *L. gracilis* Kroken ex McCune & Altermann [28]. The status of species boundaries in the genus remains unsettled. This has necessitated the recognition of two broadly defined species groups hereafter referred to as *L. columbiana* sensu lato (s.l.) [*L.’barbata’, L. gracilis, L. ‘lucida’* and *L. ‘rugosa’*] and *L. vulpina* s.l. [ = *L. v. s. str*. and *L. ‘lupina’*]. It should be noted that *L. columbiana* sensu stricto likely corresponds to one of the four provisional names applied to the fertile candidate species (*L.’barbata’, L. gracilis, L. ‘lucida’* and *L. ‘rugosa’*); however, here we follow the provisional names provided by Kroken and Taylor for consistency [18].

Understanding species boundaries in *Letharia* is particularly germane given its conservation status in Fennoscandian countries [29], [30], role in bio-monitoring research [31], [32], and role in understanding ecological and evolutionary processes in lichen symbioses [19], [20], [24], [33]. The aim of this study was to re-evaluate species boundaries and genetic structure in *Letharia* using multilocus sequence data generated from individuals collected throughout its range. Specifically, we were interested to find if complementary analytical methods and additional specimen sampling supported the same species-level lineages inferred using PSR approach for species recognition in *Letharia* [18].

Analyses based on predefined groups may obscure patterns of differentiation [34], [35] and population assignment tests provide an alternative for identifying population structure and barriers to gene flow [36]. In this study we used clustering algorithms to identify genetic groups and population structure within *Letharia* [37]–[40]. Intraspecific genetic distances were also estimated for the genetic groups inferred from the clustering analyses and compared to a recently proposed intra- interspecific threshold for Parmeliaceae [41]. We also assessed demographic histories for the inferred genetic groups to better understand evolutionary histories of *Letharia* lineages.

## Materials and Methods

### Molecular data

Molecular sequence data from a total of 432 *Letharia* specimens were included in the present study (Table S1). Our data matrix included sequences from a total of 15 markers compiled from three previous studies (Table 1; [18], [19], [42]). The 51-specimen dataset from Kroken and Taylor [18] was retrieved from TreeBase (ID# S11236) and included 13 markers: ribosomal ITS and SSU intron 287; chitin synthase I; and anonymous loci 2, 4, 11, 12, 13, 14, BA, CS, CT and DO. The 47 specimens from Högberg et al. [19] were represented by eight loci: ribosomal SSU intron 287, chitin synthase I, and anonymous loci BA, CS, CT, 4, 12, and 13. The final dataset [42], included 334 individuals, represented by up to seven loci: the ribosomal ITS and intergenic spacer (IGS); elongation factor 1-alpha (EF1-alpha); and anonymous loci BA, CS, DO, and 11. Reproductive mode and genetic variation suggest a North American origin for *Letharia* [19], [20], and 382 of the 432 specimens were collected from a total of 151 sites in North America, including a specimen from a geographically isolated *Letharia* population in the state of Veracruz, Mexico [18], [19], [42]. European populations were represented by sequences from forty-eight *Letharia* specimens collected from Italy (1 site), Norway (2 site), Sweden (4 sites), and Turkey (2 sites) [18], [19], [42]. Previous studies included *Letharia* specimens collected across an array of altitudes, substrates, and microenvironments [18], [19], [42]. The majority of sequences in the combined molecular data matrix were from specimens producing clonal propagules (i.e. soredia and/or isidioid soredia) representing the taxon *L. vulpina* s.l. [42], although a small number of *L. vulpina* s.l. specimens bore apothecia in addition to vegetative propagules. Sequences representing the fertile taxon *L. columbiana* s.l. originated from a total of 29 specimens.

**Table 1:** Genetic loci included in this study compiled from three separate studies of *Letharia*.

Locus	Length (bp)	# of variable sites	Parsimony informative sites	Primers	Source for primers
ITS† ^,^ §	554	51	27	ITS1F/ITS4A	[18]
18S intron† ^,^ ‡	388	22	15	NS26/NS18	[18]
IGS§	438	21	9	IGS12b/SSU-0072R	[86]
EF1-alpha§	515	40	27	EF-f/EF-r	[87]
Chitin synthase I† ^,^ ‡	339	13	10	chs5/chs6	[18]
BA† ^,^ ‡ ^,^ §	394	48	25	BAa/BAb	[18]
CS† ^,^ ‡ ^,^ §	339	35	25	CSa/CSb	[18]
CT† ^,^ ‡	446	28	17	CT31/CTb	[18]
DO† ^,^ §	162	13	9	DOa/DO6	[18]
2†	143	13	8	2a/2b	[18]
4† ^,^ ‡	200	12	6	4a/4b	[18]
11† ^,^ §	376	31	18	11a/CNL12	[18]
12† ^,^ ‡	140	20	12	12a/12b	[18]
13† ^,^ ‡ ^,^ §	325	21	12	13c/13d	[18]
14†	1	1	1	14a/14b	[18]

Length of individual genes alignments is given in base pairs (bp); the number of variable and parsimony informative sites is provided, along with the primers used for amplification.

† data originally reported in [18].

‡ data originally reported in [20].

§ data originally reported in [42].

### Multiple sequence alignment

Identical markers from independent studies were combined and sequences were aligned using the program MAFFT v.7 [43], [44]. For all loci, we used the G-INS-I algorithm and ‘1PAM/K = 2′ scoring matrix, with an offset value of 0.1, and the remaining parameters set to default values.

### Population clustering

We used population assignment tests implemented in the programs STRUCTURE v.2.3.2 [39], [40] and BAPS v.6 [37], [45] to identify genetic groups using all single nucleotide polymorphisms (SNPs) inferred from the aligned sequence data (Table 2). Indels and ‘N's were ignored for the purpose of SNP identification. The combined use of both methods can increase confidence in the inferred the number of clusters and population assignments when similar results are obtained independently [34]. Furthermore, both BAPS and STRUCTURE have been shown to perform well even with datasets where genetic differentiation among groups is low [34]. STRUCTURE is expected to perform well when there is sufficient independence across regions such that linkage disequilibrium within regions does not dominate the data (STRUCTURE manual), although SNP data from aligned sequence data has also been shown to effectively recover population structure [11], [46]–[48]. In contrast to the assumption of free recombination among loci in STRUCTURE, BAPS provides a linked loci-clustering model for closely linked dataset and may be more appropriate for the multilocus sequence data used in this study [49].

**Table 2:** Datasets analyzed using genetic clustering algorithms.

Dataset and source	*N*	Alignment Length (bp)	Total # of SNPs	% missing SNP data	Loci
13 loci [18]	51	3848	239	51.7	ITS, 18S intron, chitin synthase I, BA, CS, CT, DO, 2, 4, 11, 12, 13 and 14
11 loci [18]	51	3817	208	31.2	chitin synthase I, BA, CS, CT, DO, 2, 4, 11, 12, 13 and 14
6 loci [18]	51	1976	115	0.8	ITS and anonymous loci DO, 2, 11, 13, and 14
5 loci [18]	51	1424	84	1.1	anonymous loci DO, 2, 11, 13, and 14
3 loci [18]	51	1119	60	1.4	ITS and anonymous loci DO and 11
1 locus [18]	51	552	31	0.0	ITS
15 loci [18], [19], [42]	429	4759	369	41.9	ITS, 18S intron, IGS, chitin synthase I, EF1-alpha, BA, CS, CT, DO, 2, 4, 11, 12, 13 and 14

Sources for each dataset are indicated; *N*, number of included specimens; total alignment length of the concatenated alignment; the total number of single nucleotide polymorphisms (SNPs) from the concatenated alignment; the percent of missing SNP data; and the specific markers included in the analyzed dataset.

Using the same data from [18], we assessed the ability of clustering algorithms to corroborate the six species-level lineages in *Letharia* inferred under a PSR approach in [18]. First, in order to assess the impact of missing data, we compared individual population assignments between SNP data from all 13 loci (ca. 52% missing data) and a nearly complete SNP data matrix from six loci (<1% missing data) (Table 2). Second, to assess the potential dominance of the highly variable, linked ribosomal loci we excluded the ITS and SSU intron 287 and inferred population structure using SNPs from an 11-locus data matrix and a five-locus dataset excluding markers dominated by missing data (Table 2). Third, we assessed the performance of Bayesian clustering algorithms to infer population structure using sequence data from a limited number of loci. Population structure was inferred using SNPs from three markers, the ITS (the most variable locus) and two arbitrarily selected anonymous loci, DO and 11 (Table 2). We also inferred population structure based solely on SNP data from the ITS to evaluate if SNPs from a single locus could accurately infer population structure. In the STRUCTURE analyses, we implemented 10 replicate runs consisting of 50,000 burn-in generations, followed by 50,000 iterations using the admixture options for the *K* = 6 model (the number of putative species). We also explored population assignments for *K* values ranging from 1–8; with 10 replicate runs for each *K* value. Each run consisted of 50,000 burn-in generations, followed by 50,000 iterations using the admixture options. The Δ*K* method [50] was used to estimate the most likely number of clusters within the sample. Independent runs for each *K* value were combined using CLUMMP v.1.1.2 [51]. For the BAPS analysis, we used the ‘clustering with linked loci’ model, and set the upper bound for the number of populations ranging from 5–20, allowing the program to automatically infer the number of populations. Population assignments were also assessed using the ‘Fixed-K Mode’, using 100 iterations for *K* = 6. Admixture analyses across inferred genetic groups were conducted using 500 iterations, with the minimum population size taken into account when admixture is estimated set to five, up to ten reference individuals from each population, and 20 iterations for reference individuals.

We used STRUCTURE and BAPS to infer genetic structure from the combined, 15-locus dataset (*n* = 429). Clustering analyses in BAPS and STRUCTURE were performed as described above. We estimated population clusters from the combined dataset for *K* values ranging from 1–10; with 10 replicate runs for each *K* value. We used the Δ*K* method [50] to estimate the most likely number of clusters within the sample. Genetic clustering algorithms can be influenced by variation in sample sizes of distinct populations and tends to underestimate the true number of groups with unbalanced sampling [52], [53]. Sorediate *Letharia* specimens (*L*. ‘*lupina*’ and *L. vulpina*) dominated our sampling, and sample sizes of putative lineages within the apotheciate taxon *L. columbiana* s.l. were disproportionally small. We used two approaches to assess the impact of unbalanced sample sizes in our complete dataset. First, we excluded all *L. columbiana* s.l. samples and inferred genetic structure only within the sorediate *Letharia* populations. In the second approach, we reduced the number specimens representing the ‘*L. lupina*’ and ‘*L. vulpina*’ groups to 30 individuals each, similar to the number of samples representing *L. columbiana* s.l. All ‘*L*. ‘*lupina*’ and *L. vulpina* from [18] were included, and after excluding individuals with >50% missing data additional individuals were randomly selected from both the ‘*L. lupina*’ and ‘*L. vulpina*’ groups inferred from the *K* = 2 model in the STRUCTURE analysis of the sorediate specimens (see results). The randomly selected sorediate individuals were combined with the *L. columbiana* s.l. specimens. Clustering analyses of the reduced datasets were performed in BAPS and STRUCTURE, as described above.

### Phylogenetic analyses

We attempted to estimate relationships among the six putative lineages defined in [18] using the coalescent-based hierarchical Bayesian species tree model *BEAST implemented in BEAST v.1.7.5 [54], [55]. However, all exploratory analyses of the 51 OTU dataset failed to converge, even in runs implementing the simplest models, long chain lengths (>200 million generations), and data matrices without missing data. Similarly, exploratory analyses including specimens from the complete dataset (*n* = 429) also failed to converge under a number of scenarios and provided inconsistent results (data not shown). Therefore, phylogenetic relationships were estimated from the combined 15-locus dataset using a total-evidence approach [56]. Phylogenetic informativeness of sequence alignments can be visualized using likelihood mapping [57], and the phylogenetic signal from the concatenated dataset was assessed using likelihood mapping in TREE-PUZZLE v5.2 [58]. We conducted a maximum likelihood (ML) analysis of the combined data set (*n* = 429) using locus-specific model partitions in RAxML v.7.6.3 [59], [60]. A search combining 200 separate ML searches was conducted, implementing the GTRGAMMA model, and 1000 pseudoreplicates to evaluate bootstrap support for each node. ML topologies were visualized using FigTree v1.4 [61].

### Genetic distances, molecular diversity and population demographics statistics

We compared the mean pair-wise genetic distances within inferred genetic clusters to a previously identified intra- interspecific threshold in parmelioid lichens of 0.015–0.017 substitutions per site (s/s) for ITS sequences [41]. We also calculated Tajima's *D* [62] and Fu's *F* [63] statistics for the ‘*L. lupina*’ and ‘*L. vulpina*’ clusters in DnaSP, and significance was determined using the coalescent process (10,000 replicates). Under the assumption of neutrality, negative values are expected in populations that have undergone recent expansion because rare alleles are more numerous than expected, while positive values occur if rare alleles are eliminated from populations due to genetic bottlenecks or diversifying selection [62]–[64]. We did not calculate population demographic statistics for putative apotheciate lineages *L*. ‘*barbata*’, *L*. ‘*gracilis*’, *L*. ‘*lucida*’, and *L*. ‘*rugosa*’ due to small sample sizes and uncertainty of boundaries among groups.

## Results

Sample sizes, alignment lengths, numbers of SNPs, percent missing data, and included loci for each analyzed data set is reported in Table 2. The combined 15-locus dataset is deposited in TreeBase (ID# 15485). A number of sequences from the anonymous marker ‘BA’ differed substantially from previously reported variation in *Letharia*, potentially representing either paragolous gene copies or sequences of non-*Letharia* origin (e.g. algal partner or secondary fungus within the lichen thallus). The problematic BA sequences were excluded from subsequent analyses and were deposited in GenBank (accession numbers KJ610524–KJ610702).

### Clustering and population assignments

**Figure 2:**
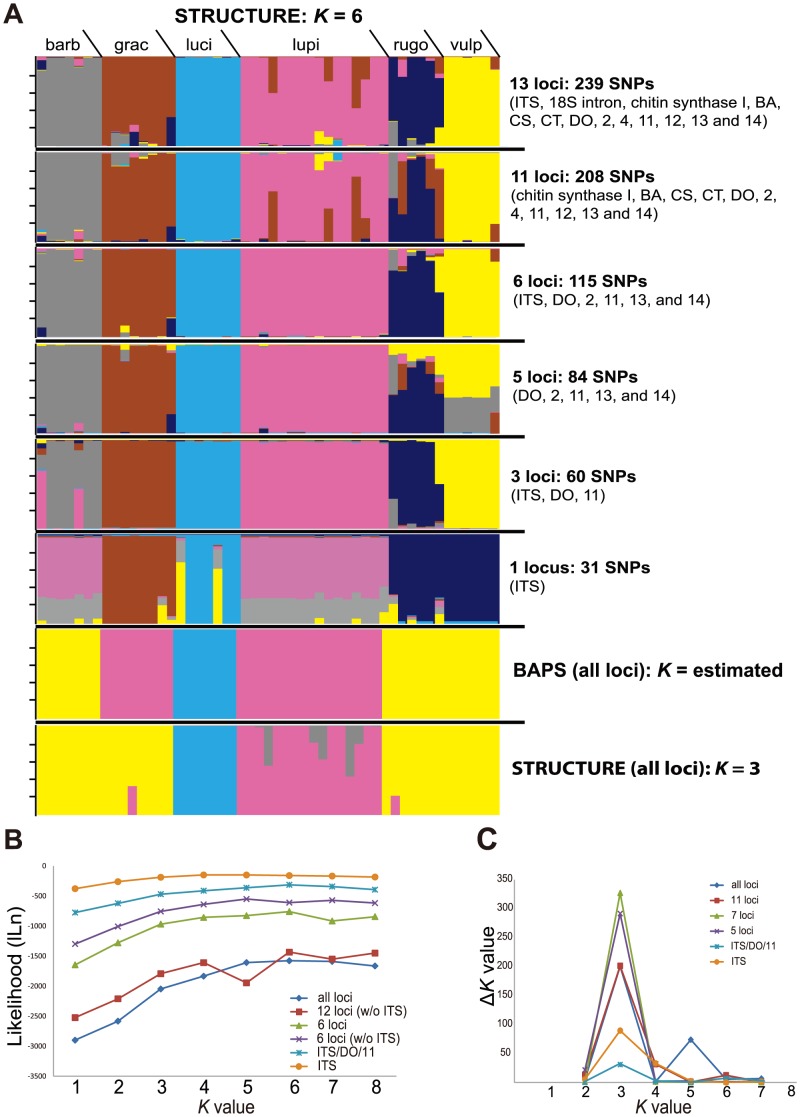
Results from Bayesian clustering analyses of 51 *Letharia* specimens from [18]. A. Individual assignments within population clusters in *Letharia* inferred using the Program BAPS and STRUCTURE; vertical bars represent individual assignment probability into different genetic clusters depicted with colors; the six uppermost panels depict individual population assignments under a variety of combinations of SNP data using a *K* = 6 model; and the lower two panels depict individual assignments inferred using BAPS (estimating the number of genetic clusters) and the *K* = 3 model from the STRUCTURE analysis (based on results from the *ad hoc* statistic Δ*K*); and order of individuals is identical across panels. B. Plot of mean likelihood values for each *K* (1–8), based on 10 replicates per *K*, from the STRUCTURE analysis of the sampled *Letharia* specimens. C. Results from the Δ*K* analysis, following Evanno et al. [51]; the modal value of this distribution is the uppermost level of structure (*K*).

Bayesian clustering of [18] original data (*n* = 51) using STRUCTURE and a *K* = 6 model supported the same six putative species-level lineages previously recognized in *Letharia*, although in a number of cases individuals showed evidence of admixed ancestry (Fig. 2A). The empirical comparisons of population assignments between a SNP data dominated by missing data (13 loci [ca. 52% missing data]) and nearly complete SNP dataset (7 loci [<1% missing data]) provided similar results (Fig. 2A). The exclusion of ribosomal data (ITS and 18S intron) had a relatively minor effect on population assignments in most cases, although individuals in the ‘*L. rugosa*’ and ‘*L. vulpina*’ groups were more frequently recovered with inferred admixed ancestries, particularly in the ‘5 loci’ dataset (Fig. 2A). The STRUCTURE analysis of SNPs from three markers (ITS, DO, 11) resulted in individual assignments consistent with the larger SNP data matrices. However, assignments based on SNP data exclusively from the ITS locus resulted in a number of differences and an increased number of individuals with inferred admixed ancestries (Fig. 2A).

Likelihood values in the STRUCTURE analyses appeared to plateau, or only have slight increases, near *K* = 3 or 4 (Fig. 2B). Rather than the six proposed species-level lineages, the Δ*K* method suggested three population clusters (Fig. 2C): one including *L*. ‘*barbata*’ (*L. columbiana* s.l.), *L*. ‘*gracilis*’ (*L. columbiana* s.l.), *L*. ‘*rugosa*’ (*L. columbiana* s.l.), and *L. vulpina* (sorediate); the second representing *L*. ‘*lupina*’ (sorediate); and the third included *L*. ‘*lucida*’ (*L. columbiana* s.l.) specimens (Fig. 2A). The BAPS analysis also inferred three distinct genetic groups similar to the three clusters from the STRUCTURE analysis, but with *L*. ‘*gracilis*’ clustering with *L*. ‘*lupina*’ rather than *L*. ‘*barbata*’, *L*. ‘*rugosa*’, and *L. vulpina* (Fig. 2A).

**Figure 3:**
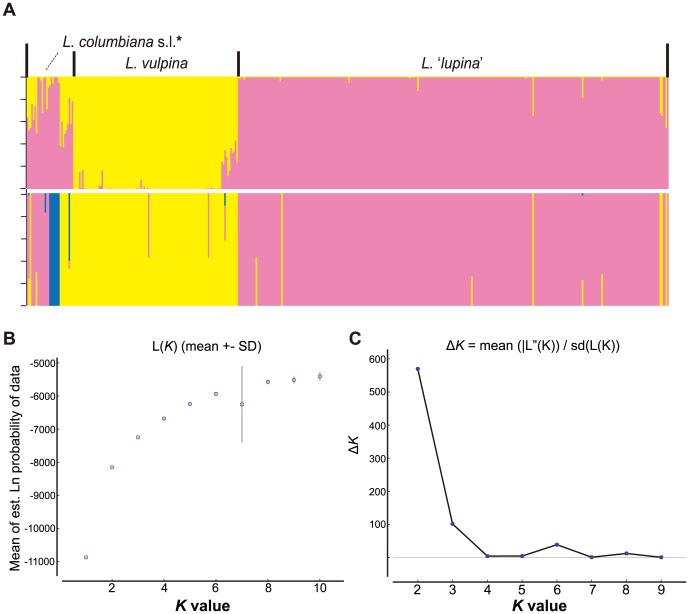
Results from Bayesian clustering analyses of 432 ***Letharia*** specimens using SNP data from a total of 15 gene regions. A. Individual ancestry within population clusters in *Letharia* inferred using the Program BAPS and STRUCTURE; vertical bars represent individual assignment probability into different genetic clusters depicted with colors; the top panel depicts individual population assignments inferred under the *K = 2* model from the STRUCTURE analysis (based on results from the *ad hoc* statistic Δ*K*); and the lower panel depict individual assignments inferred using BAPS (estimating the number of genetic clusters), where a third genetic cluster (depicted in blue) represents the putative apotheciate species *L*. ‘*lucida*.’; order of individuals is identical across panels B. Plot of likelihood values for each *K* (1–10), based on 10 replicates per *K*, from the STRUCTURE analysis of the sampled *Letharia* specimens. C. Results from the Δ*K* analysis, following Evanno et al. [51]; the modal value of this distribution is the uppermost level of structure (*K*).

In the complete 15-locus data matrix (*n* = 429), the STRUCTURE analysis inferred two distinct clusters (Fig. 3A–C). The BAPS analysis of the complete 15-locus data matrix resulted in similar population assignments, with the exception of the recovery of a third population representing the apotheciate lineage ‘*L. lucida*’ (Fig. 3A). Approximately 10% of individuals were recovered with <0.80 probability of belonging to a single cluster in the STRUCTURE analysis, including the vast majority of *L. columbiana* s.l. specimens (Fig. 3A; Table S1). The BAPS analysis recovered less than 3% of individuals with admixed genomes, which generally did not correspond to the same individuals with admixed ancestry in the STRUCTURE analysis (Fig. 3A).

**Figure 4:**
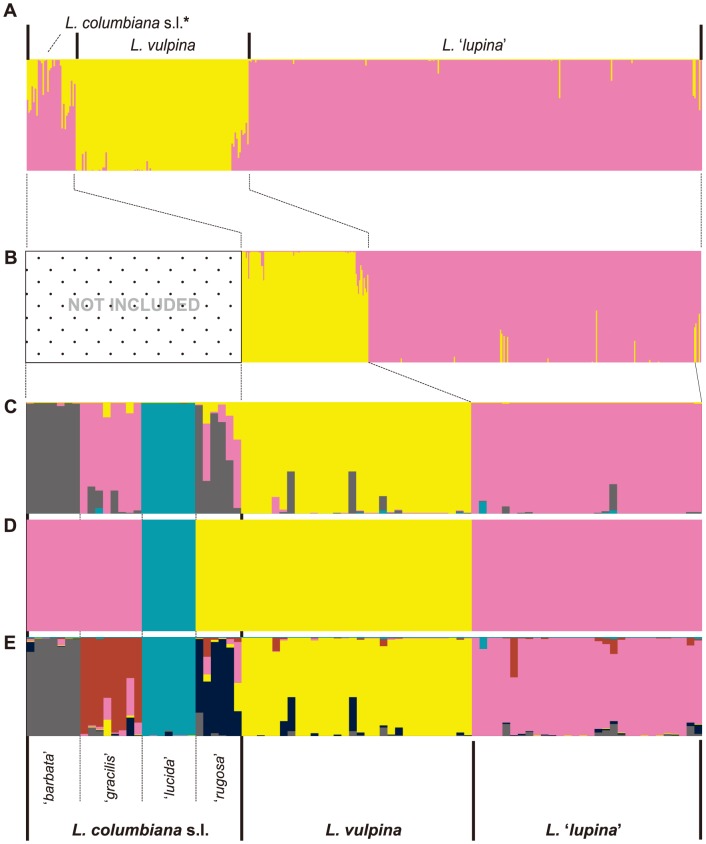
Results from Bayesian clustering analyses of ***Letharia*** samples under a variety of scenarios. Vertical bars represent individual assignment probability into different genetic clusters depicted with colors. A. Individual population assignments based on the STRUCTURE analysis of 432 *Letharia* specimens inferred under the *K = 2* model based results from the ad hoc statistic Δ*K* (identical to the top panel in Fig. 3A). B. Individual population assignments based on the STRUCTURE analysis excluding specimens representing *L. columbiana* s.l. and strictly representing sorediate forms *L*. ‘*lupina*’ and *L. vulpina*, inferred under the *K = 2* model based results from the ad hoc statistic Δ*K* (see Supplementary Fig S1). C. Individual population assignments inferred under the *K = *4 model from the STRUCTURE analysis (based on results from the ad hoc statistic Δ*K*; Supplementary Fig S2) in the dataset with normalized sample sizes for the two sorediate clusters. D. Individual assignments inferred using BAPS (estimating the number of genetic clusters) from the dataset with normalized sample sizes for the two sorediate clusters. E. Individual population assignments inferred under the *K* = 6 model from the STRUCTURE analysis (based on previous hypothesis of six species-level lineage in *Letharia*) from the dataset with normalized sample sizes for the two sorediate clusters. Order of individuals is identical across panels.

The STRUCTURE analysis of the dataset excluding *L. columbiana* s.l. specimens and strictly representing the sampled sorediate specimens clearly supported two distinct genetic clusters representing *L*. ‘*lupina*’ and *L. vulpina*, respectively (Fig. 4B; Supplementary Fig. S1). However, in the BAPS analysis a number of individuals previously recovered in the *L. vulpina* group were assigned membership in the ‘*L. lupina*’ group (Fig. S1). The analyses with normalized sample sizes for the ‘*L*. *lupina*’ and ‘*L. vulpina*’ groups, and also including *L. columbiana* s.l. specimens, provided evidence of additional substructure beyond the *K* = 2 model (Fig. 4C–D). The Δ*K* method, based on results from the STRUCTURE analysis, suggested either two or four distinct population clusters in the dataset with normalized sample sizes (Fig. 4C; Fig. S2). Assignments in the *K* = 2 model were consistent with previous assignments and are not shown. In the *K* = 4 model, ‘*L. lucida*’ and ‘*L. vulpina*’ were recovered as distinct populations; individuals previously identified as ‘*L. barbata*’ were recovered as a single population; *L. gracilis* and ‘*L. lupina*’ were recovered as a single genetic cluster; and individuals identified as ‘*L. rugosa*’ were recovered with admixed ancestry most similar to the ‘*L. barbata*’ cluster (Fig. 4C). In contrast, the BAPS analysis of the dataset with normalized sample sizes supported three distinct groups: one corresponding to the ‘*L. lupina*’ group; the seconded included the ‘*L. barbata*’, ‘*L. gracilis*’, and ‘*L. lupina*’ groups; and the third cluster included ‘*L. rugosa*’ and ‘*L. vulpina*’ (Fig. 4D). The *K* = 6 model, based on the previous hypothesis of six species-level lineages in *Letharia*, supported the same putative groups circumscribed in [18] (Fig. 4E). STRUCTURE plots for *K*>6 generally did not yield additional population clusters and individual assignments were largely consistent with the *K* = 6 model (data not shown).

### Phylogenetic signal and maximum likelihood topology

**Figure 5:**
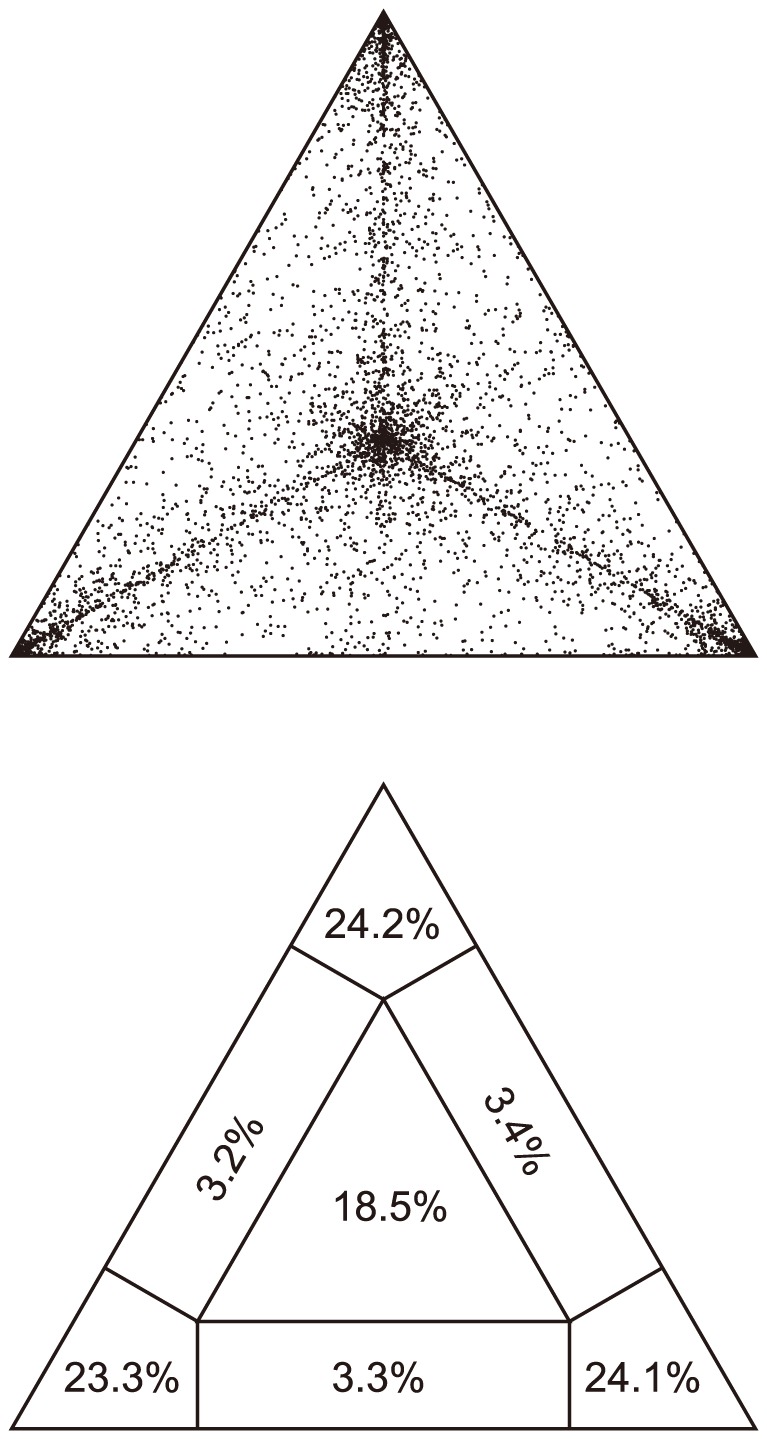
Likelihood mapping analysis of the concatenated 15-locus dataset from 432 ***Letharia*** samples. Upper panel shows the distribution pattern of all quartets and the lower panel depicts the fraction of each occupied region. The values in the panels indicated proportion of fully resolved (corners), partially resolved (along the sides), and fully unresolved quartets (in the center).

**Figure 6:**
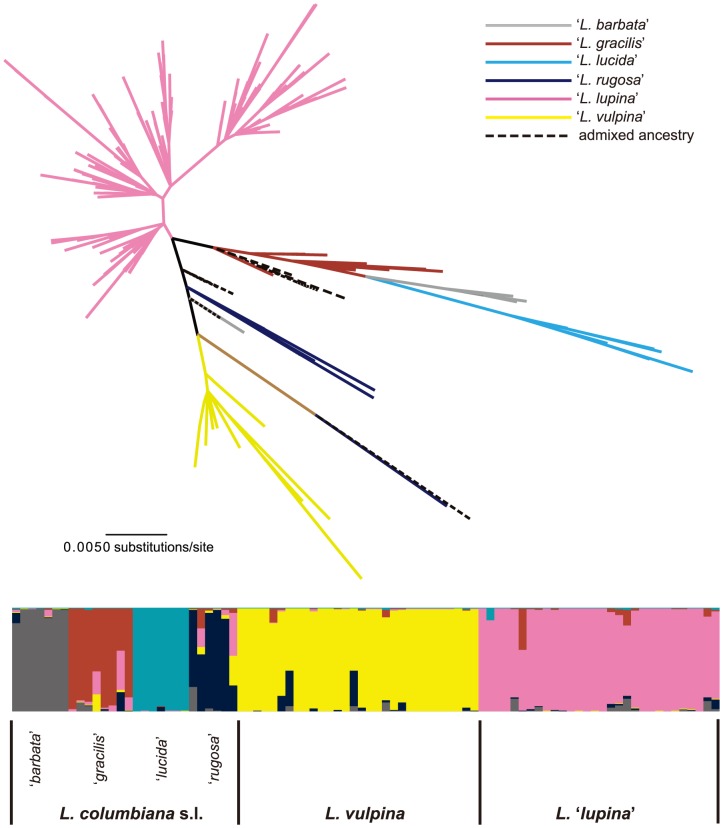
Unrooted maximum likelihood tree estimated from a concatenated 15-gene data matrix representing from 432 ***Letharia*** specimens. Colored branches correspond to population genetic clusters from the *K* = 6 model in the STRUCTURE analysis (shown in the lower panel [figure is identical to Fig. 4E]). Only ‘*L. barbata*’ and ‘*L. lucida*’ were recovered with bootstrap values >50, with bootstrap values of 69% and 100% bootstrap values, respectively.

In the likelihood mapping analysis, 71.4% of the quartets were fully resolved, 9.9% were partially resolved and 24.1% were unresolved (Fig. 5). The unrooted ML phylogeny is shown in Fig. 6. Four of the six genetic clusters – ‘*L. barbata*’, ‘*L. lupina*’, ‘*L. lucida*’, ‘*L. lupina*’ and ‘*L. vulpina*’ – from the *K* = 6 model were recovered as monophyletic; and only ‘*L. barbata*’ and ‘*L. lucida*’ were moderately to strongly supported with 69% and 100% bootstrap values, respectively (Fig. 6).

### Genetic distances and demographic histories

Pairwise genetic distances for the six candidate species are reported in Table 3. Genetic distances were generally well below the proposed intra- interspecific threshold of 0.015–0.017 substitutions/site for species in Parmeliaceae, although genetic distances for the pooled *L. columbiana* s.l. approached the intra- interspecific threshold (0.0111±0.0068). Genetic diversity indices (*H*d, S, and *π*) for the two sorediate genetic clusters – *L*. ‘*lupina*’ and *L. vulpina* are summarized in Table 4. Significant negative Tajima's D and Fu's F values were detected only in *L*. ‘*lupina*’, suggesting demographic growth for *L*. ‘*lupina*’ versus stability for *L. vulpina* (Table 4).

**Table 3:** Genetic distances among the ITS haplotypes for each candidate *Letharia* species.

Putative lineage	*N* (haplotypes)	Mean genetic distance	Range
All *Letharia* samples	376 (37/56)	0.0044±0.0042	0.0–0.0316
*L. columbiana* s.l.	28 (14/15)	0.0111±0.0068	0.0–0.0298
*L*. ‘*barbata*’	7 (4/4)	0.0016±0.0012	0.0–0.0037
*L. gracilis*	8 (3/3)	0.0014±0.0017	0.0–.0056
*L*. ‘*lucida’*	7 (3/3)	0.0062±0.0064	0.0–0.0150
*L*. ‘*rugosa*’	6 (4/5)	0.0034±0.0024	0.0–0.0074
*L*. ‘*lupina’*	280 (19/32)	0.0020±0.0017	0.0–0.0112
*L. vulpina*	59 (7/13)	0.0023±0.0044	0.0–0.0186

*N*, number of samples within each cluster (number of unique haplotypes excluding missing data/number of unique haplotypes treating missing data as a fifth character state); mean genetic distances given as the number of nucleotide substitutions per site ± standard deviation; and range of genetic distances within each putative species.

**Table 4:** Estimates of genetic diversity and drift-mutation equilibrium statistics for the ‘*L. lupina*’ and ‘*L. vulpina*’ genetic clusters in *Letharia*.

Putative lineage	*N*	*H*	*H*d	*S*	*π*	Tajima's *D*	Fu's *F*
*L.* ‘*lupina’*	280	19	0.567	22	0.00159	**−2.0407**	**−16.438**
*L. vulpina*	59	7	0.281	13	0.00221	−1.6825	−1.200

*N* =  sample size; *H* =  number of haplotypes; *H*d =  haplotype diversity; *S* =  number of segregating (polymorphic) sites; π =  nucleotide diversity; Tajima's *D*; and Fu's *F* statistic. **Bolded** values indicate significant Tajima's *D* and Fu's *F* statistic.

## Discussion

In this study we re-evaluated species boundaries in the charismatic lichen-forming fungal genus *Letharia*. While the genus has previously received considerable attention and ranks as an iconic example of cryptic diversity hidden within traditional phenotype-based species circumscriptions [18]–[20], [24], [28], [33], the number of *Letharia* species, and boundaries among them, remain unsettled. Similar to previous studies, our analyses of multilocus sequence data from 432 *Letharia* specimens collected throughout its global distribution reveal significant population structure. However, inferring the most appropriate number of genetic clusters and species-level lineages within this genus remains challenging. While our study provided additional evidence corroborating six clades [18] (Figs. 2A & 4E), the equivalence of these genetic clusters with species-level lineages is uncertain. Below we discuss some of the challenges associated with delimiting species complexes with recent diversification histories and the implications for understanding species boundaries in *Letharia.*

### Delimiting species at the interface of traditional population genetic and phylogenetic analyses

Our data suggest a recent diversification history for *Letharia*. The 15-gene ML phylogeny of the genus generally did not recover well-supported phylogenetic substructure indicative of long-term reproductive isolation among lineages (Fig. 6). Similarly, relationships in individual gene topologies were unresolved (data not shown) and the likelihood mapping analysis revealed only moderate phylogenetic signal in the 15-gene data matrix (Fig. 5). Although a lack of phylogenetic structure may also indicate historic or ongoing gene flow among lineages rather than a recent diversification history, estimates of ITS pairwise genetic distances for the entire genus fell below a proposed intra- interspecific threshold of 0.015–0.017 substitutions/site for species in Parmeliaceae (Table 3; [41], suggesting a recent origin of all extant *Letharia* lineages. The estimated pairwise distances for *Letharia* are similar to a species complex in *Xanthoparmelia* (Parmeliaceae) with a diversification history apparently coinciding with climatic fluctuations during the Pleistocene [47], [65].

While phylogenetic inferences can provide valuable insight into species boundaries, particularly in cases of deeper divergence histories, delimiting species with more recent diversification histories remains challenging (e.g. [66], [67]). A number recently developed coalescent-based species tree methods may accurately reconstruct relatively shallow divergence histories from multilocus sequence data, even with moderate levels of recombination [68], [69]. However, most of these methods require that species/population assignments are made *a priori* [10], [55], [70], [71]. Furthermore, most currently available coalescent-based methods are dependent on the quality of individual gene estimates and do not account for hybridization [12], [72], [73]. Our attempts to estimate relationships among the inferred genetic groups in *Letharia* failed to converge to a stable, stationary distribution. This may be the result of excessive splitting of populations or levels of genetic variation that are too low to accurately estimate parameter values. Similarly, relationships in the ML topology were recovered with weak statistical support (Fig. 6).

The recently developed Bayesian species delimitation program Bayesian Phylogenetics and Phylogeography (BPP; [12], [74]) provides an objective approach for delimiting species boundaries in closely related species complexes, and has recently been shown to perform accurately using a modest number of genetic markers in cases of recent speciation [13]. However, BPP requires a fully resolved guide tree, and misspecifications of the guide tree can result in strong posterior support for models containing more species [75]. Ultimately, we were unable to obtained a well-supported guide tree required for the Bayesian species delimitation program BPP.

### Bayesian clustering using SNPs from multi-locus sequence data

Due to the recent divergence history of *Letharia* and uncertainties in specimen identification, we used population assignment tests in the programs BAPS and STRUCTURE to characterize genetic differentiation among populations without *a priori* specimen assignments. Both programs have been shown to accurately infer the number of subpopulations, even when genetic differentiation is low [34]. Previous studies have shown that SNP data from aligned sequence data can effectively recover population structure [11], [47]–[49], [76], [77]. Using SNPs from the 13-locus data set (*n* = 51) reported in [18], we show that Bayesian clustering implemented in the program STRUCTURE was generally able to recover the same six putative *Letharia* lineages circumscribed using a gene genealogical approach (Figs. 2A & 4E; [18]. Population assignments were largely consistent across a range of scenarios, with extensive amounts of missing data, exclusion of SNPs from variable markers (ITS and 18S intron), and inferences based on SNPs from as few as three gene regions (Fig. 2A). The overall corroboration between the results of the STRUCTURE analyses and the previously circumscribed species-level lineages further indicates that SNPs from multilocus sequence data can accurately recover population structure, even using data sets with missing data or relatively few gene regions.

One of the major challenges to population assignment tests is the ability to detect the most appropriate number of genetic clusters (*K*) [34], [37], [51], [78]. Although estimating the true value of *K* may be difficult, the ad hoc statistic Δ*K*, based on the rate of change in the log probability of data between successive *K* values in STRUCTURE runs, has been shown to provide an accurate perspective on the uppermost hierarchical level of structure in some cases [51]. Similarly, [40] recommended identifying the smallest value of *K* that captures the major structure of the data and seems biologically sensible. Although our results were congruent with the previous hypothesis of species boundaries in *Letharia* when assuming *K* = 6, both the BAPS analysis and the *ad hoc* statistic Δ*K* from results of the STRUCTURE analysis [51] suggest that population structure is likely captured with fewer genetic groups (Fig. 2 & Fig. 3). Our data provide evidence that species-level genetic clusters may include both the apotheciate *L. columbiana* s.l. and *L. vulpina* s.l. The *ad hoc* Δ*K* approach for assessing results from the STRUCTURE analyses of [18] original data (*n* = 51) supported three distinct genetic groups (Fig. 2C), lumping *L. vulpina* with all *L. columbiana* s.l. specimens (Fig. 2A). Similarly, the BAPS analyses recovered three of the four putative *L. columbiana* s.l. species within either the ‘*L. lupina*’ or *L. vulpina* genetic clusters (Figs. 2A & Fig. 4D). In the *K* = 4 model based on the normalized sampling of populations, samples representing the apotheciate taxon ‘*L. gracilis*’ were frequently assigned to the same genetic cluster containing ‘*L. lupina*’ specimens (Fig. 4C).

The inference of fewer than six distinct genetic groups in *Letharia* may be an artifact of unbalanced sample sizes (Fig. 4), and our findings highlight the potential for different inferences of population substructure under unbalanced sampling schemes [53], [54], [79]. In this study, we attempted to balance sample sizes among putative groups by either excluding populations with small sample sizes (i.e. *L. columbiana* s.l.) (Fig. 4B) or normalizing sample sizes across groups (Fig. 4C–E). We identified evidence supporting additional population substructure in both scenarios. Clustering analyses either excluding populations with small sample sizes (i.e. *L. columbiana* s.l.) or normalizing sample sizes across groups resulted in varying inferences of *K* (Fig. 4B–E). While the apotheciate specimens previously identified as ‘*L. lucida*’ were consistently supported as a distinct genetic group, the number of genetic clusters and relationships of the putative apotheciate lineages (‘*L. barbata*’, ‘*L. gracilis*’ and ‘*L. rugosa*’) and sorediate groups (*L. lupina* and *L. vulpina*) remain largely ambiguous (Fig. 4C–E). Regardless, our data suggest that unbalanced sample sizes likely have a greater impact on inferring the appropriate number of populations than missing data. Population assignments of individuals were consistent between analyses including missing data and others excluding loci dominated by missing data (Fig. 2A), but differed significantly with varying sizes of sampled populations (Fig. 4).

Although both BAPS and the Δ*K* method support fewer than six genetic clusters, this study provides corroborating evidence that the six previously circumscribed *Letharia* species do in fact represent some level of genetically isolated groups [18]. Under a *K* = 6 model, individual assignments were largely consistent across a range of different combinations of data (Fig. 2A), suggesting that the six candidate species from [18] are not simply the result of stochastic signal in individual loci. Furthermore, subtle morphological differences have been identified for most of the six putative *Letharia* species [18], [28]. Our study provides compelling evidence supporting two distinct sorediate populations in *Letharia*, *L. vulpina* and *L.* ‘*lupina*’ (Figs. 2, 3, & 4). Similarly, the apotheciate group *L.* ‘*lucida*’ is generally supported as a distinct genetic cluster (Fig. 4C–E) and is the only putative species recovered with strong support in the ML phylogeny (BS = 100; Fig. 6). *Letharia gracilis* has recently been formally described based on differences in branching morphology and distinct ITS sequence variation [28]. However, additional sampling of *L. columbiana* specimens throughout their distribution in western North America will be required to fully resolve relationships among putative apotheciate lineages.

### Biogeography and population demographics

In *Letharia*, the sorediate groups, *L*. ‘*lupina*’ and *L. vulpina*, have a much broader geographic distribution than *L. columbiana* s.l. *Letharia* ‘*lupina*’ is widely distributed across western North America, where it is more commonly encountered than *L. vulpina*; and also occurs in the state Veracruz, Mexico, Switzerland, and has been reported from Morocco [20]. *Letharia vulpina* is the far more common species occurring in continental Europe, and has been found in Cyprus and the Caucasus [19], [20]. In North America, *L. vulpina* generally occurs in warmer coastal mountains near the Pacific Coast, in contrast to *L*. ‘*lupina*’ which is the most common species in the interior western mountains – the Sierra Nevada, Cascades, and Rocky Mountains [80], although both lineages occur sympatrically in some areas. While previous research has further documented the occurrence of *L*. ‘*lupina*’ in the Old World [20], sequences from this study were not available and therefore not included in our current study.

While it has previously been suggested that *Letharia* populations in central and Northern Europe were re-colonized from refugial populations in Caucasus and Morocco, our data failed to provide evidence supporting demographic expansion in *L. vulpina* (Table 4). Previous research suggests that Pleistocene glacial cycles were not inherently unfavorable or restrictive for some high altitude/latitude lichen-forming fungal species [80]–[82], and our data suggest that *L. vulpina* has maintained a relatively stable population size. In contrast, *L*. ‘*lupina*’ appears to have experienced recent demographic growth (Table 4). Although the contrasting demographic histories in the two sorediate clusters in *Letharia* remain unexplained, it may be due, in part, to differences in associations with the obligate algal partner. *Letharia vulpina* has been shown to associate with a unique algal clade, provisionally named *Trebouxia* ‘*vulpinae*’, while *L.* ‘*lupina*’ associates with other clades within *Trebouxia* [83]. Whether these interactions are the result of biogeographic availability, increased fitness, or genetic factors remains unexplained. Ultimately, additional research will be required to elucidate factors influencing demographic histories in *Letharia.*

### Conclusions

Species delimitation remains a contentious issue in most biological groups [6], [14]. While the application of a wide range of species delimitation analyses may increase confidence in well circumscribed species [2], our ability to detect boundaries among lineages in recent radiations is limited to a small subset of operational criteria. Currently, the most appropriate number of genetic groups and their equivalence to species-level lineages remain difficult to objectively define in *Letharia*.

Nevertheless, our study consistently supported two distinct sorediate groups, ‘*L. lupina*’ and *L. vulpina* (Figs. 2, 3 & 4), and subtle morphological differences support this distinction (Altermann et al. *in revision*). Similarly, the putative apotheciate species ‘*L. lucida*’ was also consistently supported as a distinct group in the analyses of normalized sample sizes across groups (Fig. 4). However, additional studies will be required to elucidate the relationship of the closely related apotheciate populations – *L. columbiana* s.l., including the recently described *L. gracilis* [28] – to the two sorediate clusters. Outcrossing and recombination have been shown to be common in both fertile and generally sorediate *Letharia* populations [24]. Closely related species in rapidly diversifying radiations may be particularly prone to hybridization [84], [85], and reproductive barriers among closely related *Letharia* populations remain unknown. Population genomic studies using high-throughput sequencing data have been shown to provide important insight into recent radiations (ex. [17]) and will likely prove valuable for understanding the evolutionary history of *Letharia*. Results from our study provide a framework for future studies investigating species boundaries in this genus.

## Supporting Information

Figure S1 **Individual ancestry within population clusters in** ***Letharia*** **inferred using the Program BAPS and STRUCTURE based exclusively on sorediate specimens** ***L. ‘lupina*** **’ and** ***L. vulpina*** **.** A. Individual population assignments inferred in the STRUCTURE analysis excluding specimens representing *L. columbiana* s.l. and strictly representing sorediate forms *L*. ‘*lupina*’ and *L. vulpina*, inferred under the *K = 2* model based results from the ad hoc statistic Δ*K*. B. Individual population assignments inferred in the BAPS analysis excluding specimens representing *L. columbiana* s.l. and strictly representing sorediate forms *L*. ‘*lupina*’ and *L. vulpina*, C. Plot of likelihood values for each *K* (1–10), based on 10 replicates per *K*, from the STRUCTURE analysis of the sampled sorediate *Letharia* specimens. C. Results from the Δ*K* analysis, following Evanno et al. [51]; the modal value of this distribution is the uppermost level of structure (*K*).
(PDF)

Figure S2 **Likelihood and Δ** ***K*** **plots from the STRUCTURE analysis of the dataset with normalized sample sizes.** A. Plot of likelihood values for each *K* (1–10), based on 10 replicates per *K*, from the STRUCTURE analysis of the sampled sorediate *Letharia* specimens. B. Results from the Δ*K* analysis, following Evanno et al. [51]; the modal value of this distribution is the uppermost level of structure (*K*).
(PDF)

Table S1 **Collection and sequence information for all** ***Letharia*** **specimens included in the present study**. GenBank accession numbers for sequences >200 base pairs (bp) are provided for sequences from Altermann [42]; sequences <200 bp are available in TreeBase under study identification number 15485 (listed here as ‘TB S15485’). Previously reported sequences from Kroken and Taylor [18] are available in TreeBase under study identification number 11236 (listed here as ‘TB S11236’); sequences reported previously in Högberg et al. [19] are available in TreeBase study # 15485 (listed here as ‘TB S15485*'). **Bolded** GenBank accession numbers for the ‘BA’ locus represent sequences that were excluded from clustering analyses (see text).
(XLSX)
