# Supplementary figures and images for: How Do You Solve a Problem like Letharia? A New Look at Cryptic Species in Lichen-Forming Fungi Using Bayesian Clustering and SNPs from Multilocus Sequence Data
Source: PLoS One. 2014 May 15;9(5):e97556. doi: 10.1371/journal.pone.0097556 (PMC4022584; doi:10.1371/journal.pone.0097556)

**A****STRUCTURE  $K = 2$** 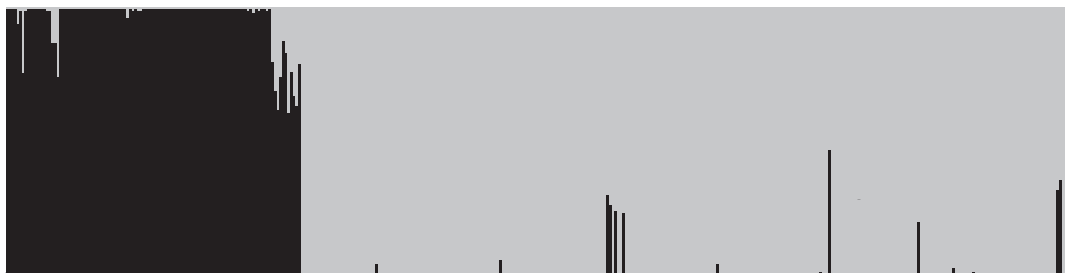**B****BAPS estimated  $K$** 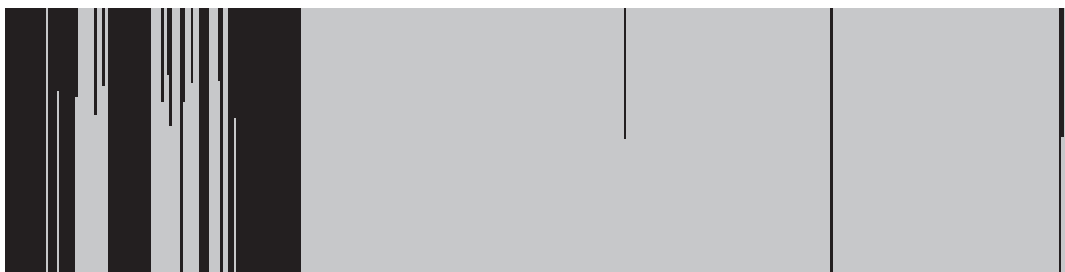**C** **$L(K)$  (mean  $\pm$  SD)**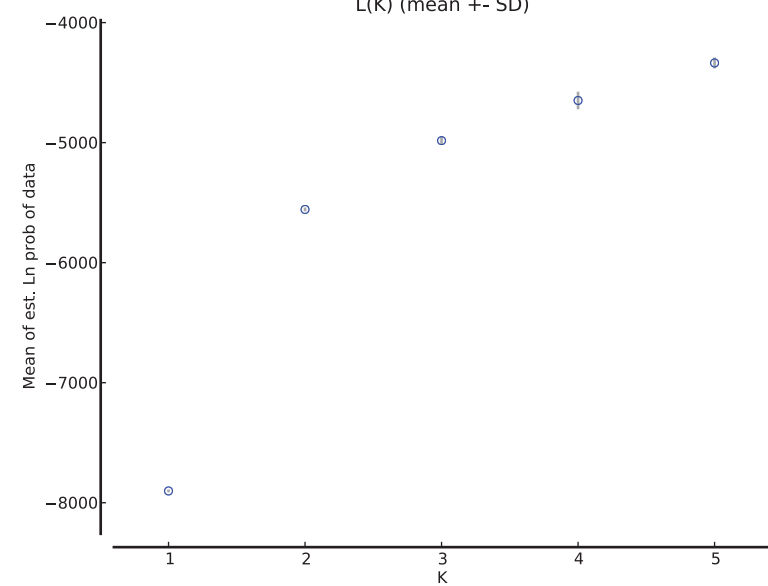**D** **$\Delta K = \text{mean}(|L''(K)|) / \text{sd}(L(K))$** 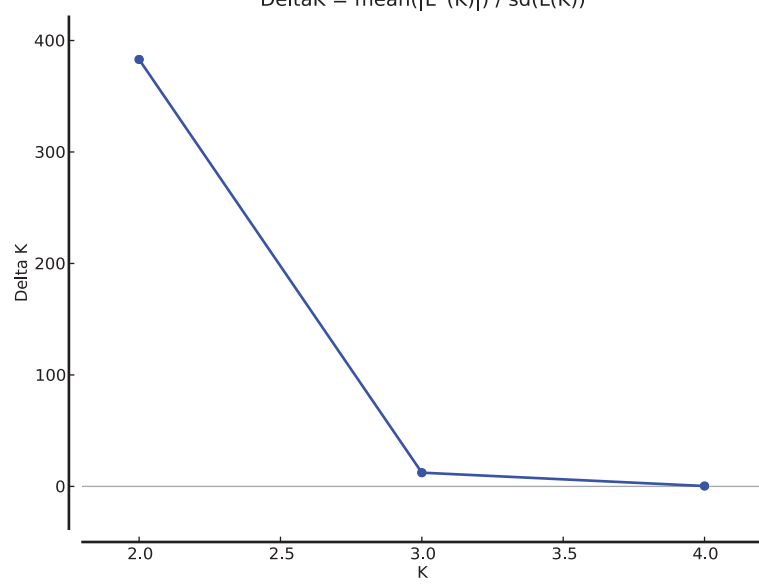

Supplement: Figure S1 — Individual ancestry within population clusters in Letharia inferred using the Program BAPS and STRUCTURE based exclusively on sorediate specimens L. ‘lupina ’ and L. vulpina . A. Individual population assignments inferred in the STRUCTURE analysis excluding specimens representing L. columbiana s.l. and strictly representing sorediate forms L. ‘lupina’ and L. vulpina, inferred under the K = 2 model based results from the ad hoc statistic ΔK. B. Individual population assignments inferred in the BAPS analysis excluding specimens representing L. columbiana s.l. and strictly representing sorediate forms L. ‘lupina’ and L. vulpina, C. Plot of likelihood values for each K (1–10), based on 10 replicates per K, from the STRUCTURE analysis of the sampled sorediate Letharia specimens. C. Results from the ΔK analysis, following Evanno et al. [51]; the modal value of this distribution is the uppermost level of structure (K). (PDF) [file pone.0097556.s001.pdf]

A

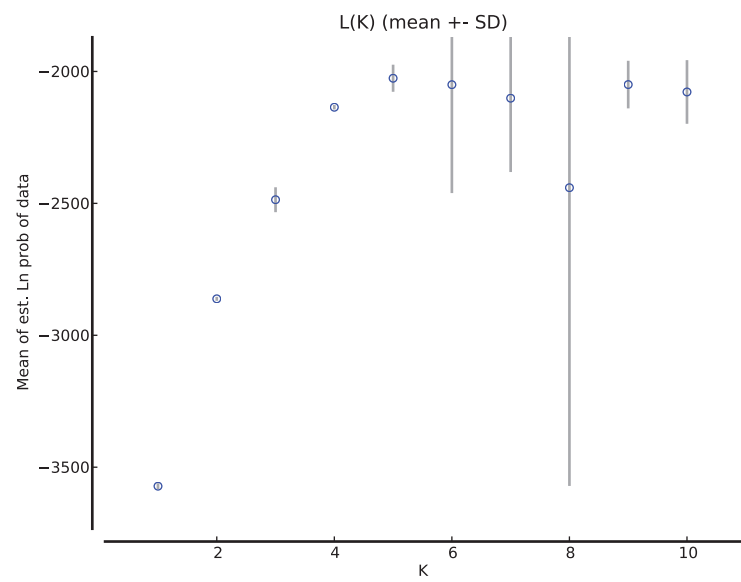

B

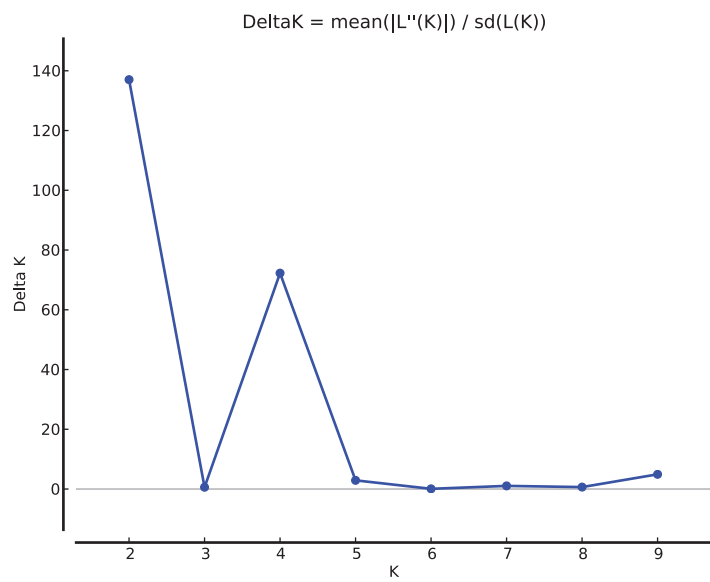

Supplement: Figure S2 — Likelihood and Δ K plots from the STRUCTURE analysis of the dataset with normalized sample sizes. A. Plot of likelihood values for each K (1–10), based on 10 replicates per K, from the STRUCTURE analysis of the sampled sorediate Letharia specimens. B. Results from the ΔK analysis, following Evanno et al. [51]; the modal value of this distribution is the uppermost level of structure (K). (PDF) [file pone.0097556.s002.pdf]
